# A Descriptive, Retrospective Analysis of COVID-19 Passive Antibody Therapy and Its Effects on Morbidity and Mortality in Patients Receiving B-Cell-Depleting Therapies

**DOI:** 10.3390/diseases12020033

**Published:** 2024-02-06

**Authors:** Sonia Gentile, Liam R. Sullivan, Heather Brooks, Gordana Simeunovic

**Affiliations:** 1 Department of Internal Medicine and Pediatrics, Corewell Health, Grand Rapids, MI 49503, USA; 2 College of Human Medicine, Michigan State University, Grand Rapids, MI 49503, USA; 3 Department of Infectious Disease, Corewell Health, Grand Rapids, MI 49503, USA; 4 Office of Research and Education, Corewell Health, Grand Rapids, MI 49503, USA

**Keywords:** COVID-19, monoclonal antibody, convalescent plasma, immunosuppression, rituximab

## Abstract

Patients receiving B-cell-depleting therapies (BCDT) are at an increased risk for severe COVID-19. Passive antibody therapy (PAT), including COVID-19 convalescent plasma (CCP) and monoclonal antibodies (mAb), may be an effective treatment in this population. Real-world data on PAT effectiveness are limited. To evaluate response to PAT measured through 90-day all-cause morbidity and mortality, we performed a retrospective review of patients who contracted COVID-19 within a year from the last BCDT. From 64 included patients, the majority were Caucasians (95%), female (56%), vaccinated (67%), treated outpatients (64%), with multiple comorbidities. Examined BCDT were rituximab (55%), obinutuzumab (33%), ocrelizumab (11%) and ofatumumab (1%), used for underlying hematological malignancy (HEM) (40%), multiple sclerosis (34%), and rheumatoid arthritis (16%). Of seven deceased patients, three died from COVID-19. All three were elderly males with multiple comorbidities, treated inpatient for severe COVID-19. Four of 41 patients treated as outpatients were hospitalized for non-COVID-19-related reasons. All deceased and hospitalized patients had an underlying HEM. All but one were on rituximab. PAT may be an effective treatment for patients receiving BCDT, especially if given early for non-severe disease. Patients with underlying HEM may be at increased risk for severe disease compared with others receiving the same BCDT.

## 1. Introduction

The COVID-19 pandemic has caused significant morbidity and mortality worldwide with an estimated mortality rate of 1.1% in the general population of the United States of America [1]. Immunocompromised patients are at an increased risk of severe COVID-19 infection. This particularly applies to patients with underlying autoimmune diseases, hematologic malignancies (e.g., B-cell lymphomas or B-cell lymphocytic leukemias), and neurologic disorders who are being treated with B-cell-depleting therapies.

B-lymphocytes differentiate into either memory B-cells or plasma cells upon antigen exposure. Memory B-cells are precursors to antibody-producing cells and serve as antigen-presenting cells through interactions with CD4 T lymphocytes that recognize the same antigen [2]. Anti-CD20 monoclonal antibodies are frequently used as B-cell-depleting therapies. Commonly used anti-CD20 monoclonal antibodies include agents such as rituximab and obinutuzumab. Treatment with anti-CD20 monoclonal antibodies results in complete B-cell depletion within 72 h (about 3 days), with an estimated recovery time of 6 to 9 months after the completion of therapy and a return to normal levels after 9 to 12 months. Severe B-cell depletion compromises the ability of the immune system to make antibodies, such as neutralizing antibodies, which are important for the clearance of many infections, including respiratory viruses such as SARS-CoV-2. Depletion of neutralizing antibodies can significantly increase a patient’s risk for severe infections, including COVID-19 infection [3].

Studies of COVID-19 infection in patients receiving B-cell-depleting therapies have also shown an inadequate response to COVID-19 vaccination, a more severe and protracted disease course, and poor clinical outcomes [4,5,6]. The standard of care treatment of COVID-19 infection in the general population includes antiviral agents, anti-inflammatory agents, and passive antibody therapies, including monoclonal antibodies and COVID-19 convalescent plasma. Monoclonal antibodies imitate natural monoclonal IgG antibodies and bind non-competitively to the SARS-CoV-2 spike protein receptor, blocking the ability of the virus to enter human cells [7]. Monoclonal antibodies have been shown to be effective in immunocompetent patients with mild to moderate COVID-19 infection who are not hospitalized [8,9]. COVID-19 convalescent plasma from donors who have recovered from COVID-19 infection may contain high levels of neutralizing antibodies to SARS-CoV-2 that could help suppress viral replication, enhance viral clearance, and prevent progression of COVID-19 infection from mild to moderate or severe disease [7]. COVID-19 convalescent plasma containing anti-SARS-CoV-2 antibodies from the COVID-19 infection survivors has also been shown to be beneficial for certain groups of patients [10,11].

Patients receiving B-cell-depleting therapies have difficulty in producing neutralizing antibodies against all viruses, including SARS-CoV-2. Thus, it is reasonable to hypothesize that, theoretically, passive antibody therapies, including both monoclonal antibodies and COVID-19 convalescent plasma, may be effective in treating COVID-19 infection in this patient population. Several small case series, systematic reviews and meta-analysis have suggested a benefit of use of passive antibody therapy in this group of patients [12,13,14,15,16]. However, convincing evidence is still missing because of the lack of prospective randomized placebo-controlled trials. A better understanding of the efficacy of passive antibody therapies in immunocompromised patients can help optimize COVID-19 treatment in this patient population in the future, direct future research related to COVID-19 treatment, and potentially be beneficial in the development of new treatments for other viral infections. The goal of this study is to describe additional real-world evidence supporting the potential efficacy of passive antibody therapies in the treatment of immune-suppressed COVID-19-infected patients who received B-cell-depleting therapies within the previous year by evaluating the 90-day all-cause morbidity and mortality.

## 2. Materials and Methods

### 2.1. Setting and Cohort

This is a retrospective, descriptive single-center study of 64 patients receiving B-cell-depleting therapies who contracted COVID-19 infection within one year from the last administration of a B-cell-depleting therapy and who subsequently received treatment with COVID-19 passive antibody therapies, including monoclonal antibodies or COVID-19 convalescent plasma between 1 April 2020 and 5 July 2022.

Corewell Health West is a nonprofit quaternary-care health system based in Grand Rapids, Michigan that offers care to patients through fourteen hospitals and one hundred and fifty ambulatory clinics. As a part of the response to the COVID-19 Public Health Emergency, in December 2020, Corewell Health West established a COVID-19 Treatment Clinic. The role of the COVID-19 Treatment Clinic was to coordinate COVID-19 treatments systemwide, both inpatient and outpatient. The Food and Drug Administration issued an Emergency Use Authorization for multiple different monoclonal antibodies with the recommendation to use them for treatment of mild to moderate COVID-19 disease in non-hospitalized patients who were at high risk for disease progression [17,18,19]. COVID-19 convalescent plasma received Emergency Use Authorization for treatment of immunocompromised hospitalized patients infected with COVID-19 [20]. Under special circumstances, when COVID-19 convalescent plasma was not available, we were able to obtain monoclonal antibody therapy for hospitalized patients under compassionate single-patient investigational new drug application (IND) with special approval from our local Institutional Board Review, monoclonal antibodies manufacturer, and the Food and Drug Administration. Under the guidance of the COVID-19 Treatment Clinic, COVID-19 positive patients receiving B-cell-depleting therapies were treated with either monoclonal antibodies or COVID-19 convalescent plasma, depending on the patient location and treatment availability. Per the Emergency Use Authorization guidelines and Institutional Review Board requirements, all patient-related data were collected and stored in the COVID-19 Treatment Clinic database. We compiled data from the COVID-19 Treatment Clinic database to evaluate various patient characteristics and accessed patient Electronic Medical Records to ascertain outcomes after treatment with monoclonal antibodies or COVID-19 convalescent plasma.

**Figure 1:**
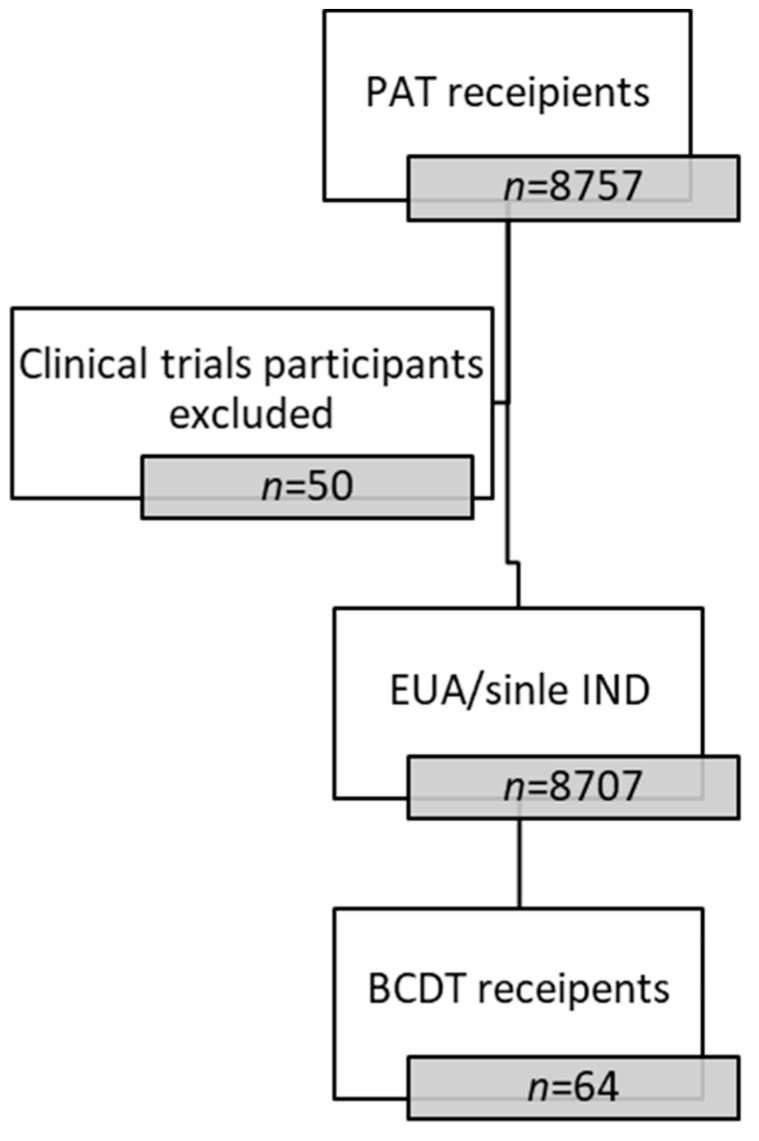
Patient Flow Diagram. PAT = passive antibody therapy. EUA = emergency use authorization. BCDT = B-cell-depleting therapy.

Between 1 April 2020, and 5 July 2022, 8757 patients received passive antibody therapy in our COVID-19 Treatment Clinic. Out of 8757 patients, fifty patients were excluded due to participation in clinical trials. Of the remaining 8707 patients who received passive antibody therapies under the Emergency Use Authorization or via compassionate single-patient IND, sixty-four patients were identified to have been on treatment with B-cell-depleting therapies within the year prior to contracting COVID-19 and were included in this analysis (Figure 1).

### 2.2. Outcomes

We evaluated two primary outcomes: all-cause 90-day mortality and all-cause 90-day morbidity. All-cause 90-day mortality was defined as death within 90 days from passive antibody therapy administration. We first evaluated all-cause 90-day mortality for the whole cohort. We then divided patients into two groups based on infusion location (outpatient versus inpatient) and evaluated patient characteristics and all-cause 90-day mortality in each group. We also evaluated patient characteristics based on the all-cause 90-day mortality and compared the characteristics of survived and deceased patients. The cause of death was determined based on the charted cause of death and confirmed by the independent review of two investigators. All-cause 90-day morbidity was defined as at least one hospitalization within 90 days from passive antibody therapy administration in patients treated in the outpatient setting. Binary variables were created for each outcome as the presence or absence of at least one hospital admission for patients treated in the outpatient setting, as well as the presence or absence of death for all patients.

### 2.3. Covariates

We collected the following variables from the COVID-19 treatment clinic database: general patient demographics including age, gender, race, comorbidities representing the risk factors for disease progression, as listed under the Emergency Use Authorization, number and type of COVID-19 vaccines received, presence or absence of COVID-19 anti-bodies, underlying diagnosis requiring treatment with B-cell-depleting therapy, type and date of the last B-cell-depleting therapy, date of COVID-19 infection symptom onset, date of positive COVID-19 test, date of passive antibody therapy administration, and type of passive antibody therapy received. We collected the following variables from the electronic medical records: dates of hospital admission and discharge, reason for hospital admission, COVID-19 PCR result including cycle threshold if available, date of admission and discharge from the intensive care unit, the highest level of respiratory support during hospitalization, type and duration of any COVID-19 treatments administered during hospital stay, date of death, and cause of death. Electronic medical records of deceased patients were evaluated independently by two investigators to confirm the cause of death. Patients were considered vaccinated if they previously received two doses of Pfizer or Moderna vaccines, or a single dose of Johnson and Johnson vaccine. We categorized the variables in the following way: patient sex (male or female), age (continuous), race (non-Hispanic White, Black or African-American, Hispanic, Other), comorbidities (the type and number of comorbidities), indication for B-cell-depleting therapies (hematological malignancies, multiple sclerosis, rheumatoid arthritis and others that included granulomatosis with polyangiitis, granulomatous interstitial lung disease, systemic sclerosis, and systemic lupus erythematosus), type of B-cell-depleting therapy (rituximab, obinutuzumab, ocrelizumab, and ofatumumab), COVID-19 vaccination status (vaccinated or unvaccinated), COVID-19 antibody status (positive or negative), time from the symptom onset to passive antibody therapy administration (0 to 7 days, and further in 7-day increments), passive antibody therapy infusion location (outpatient or inpatient), type of passive antibody therapy administered (monoclonal antibodies, including casirivimab–imdevimab, sotrovimab, bamlanivimab–etesevimab and bebtelovimab, or COVID-19 convalescent plasma), type of additional COVID-19 treatments (Paxlovid, remdesivir, corticosteroids, tocilizumab), cause of death (COVID-19-related or not COVID-19-related).

### 2.4. Statistical Analysis

We performed a descriptive analysis using means and frequencies (percentage). Absolute risk was calculated for clinical outcomes: all-cause 90-day mortality and all-cause 90-day morbidity, by dividing the number of patients with the outcome by the total number of eligible patients. All categorical variables are displayed as count (percentage) and tested using Chi-Square.

## 3. Results

### 3.1. Cohort Description

Our study included sixty-four patients, the majority of whom were female (56%) and non-Hispanic Caucasian (95%). The mean age was 56.6 years. Sixty-seven percent of our patients were vaccinated. COVID-19 serology status was unknown for most of our patients and was not included in the final analysis. Indication for B-cell-depleting therapies included hematological malignancies (26/64, 40%), multiple sclerosis (22/64, 34%), rheumatoid arthritis (10/64, 14%) and others (6/64, 11%). Among the six patients grouped under “others”, three had a diagnosis of granulomatosis with polyangiitis (*n* = 3), one granulomatous interstitial lung disease, one systemic sclerosis, and one systemic lupus erythematosus. Seventy-seven percent of patients had at least one additional comorbidity listed as a risk factor for severe COVID-19 infection. Evaluated B-cell-depleting therapies included rituximab (35/64, 55%), obinutuzumab (21/64, 33%), ocrelizumab (7/64, 11%), and ofatumumab (1/64, 1%). Seventy-seven percent of all patients (49/64) were treated with monoclonal antibodies: casirivimab–imdevimab (*n* = 31), sotrovimab (*n* = 13), bamlanivimab–etesevimab (*n* = 4) and bebtelovimab (*n* = 1), while 23% of patients (*n* = 15) received COVID-19 convalescent plasma (Table 1).

Sixty-four percent (41/64) of our patients were treated in the outpatient setting, while 36% (23/64) received treatment upon admission to the hospital for severe COVID-19 infection. Compared with the patients who received passive antibody therapies during their hospitalization for COVID-19, patients treated in the outpatient setting were on average younger (51.4 vs. 65.9), more frequently vaccinated (80% vs. 43%, *p* = 0.0025), and had fewer comorbidities. Patients treated in the outpatient setting were more likely to be treated earlier in the course of the disease (*p* < 0.0005). The majority of patients treated in the outpatient setting had underlying Multiple sclerosis (51%), followed by hematological malignancies (22%) and rheumatoid arthritis (17%), while 74% of hospitalized patients had an underlying hematological malignancy (*p* = 0.0093). Hospitalized patients were more likely to receive rituximab for their underlying disease (74% vs. 44%, *p* = 0.0013). All patients treated in the outpatient setting received monoclonal antibodies, compared with only 35% of patients treated while inpatient; the remaining 65% of hospitalized patients received COVID-19 convalescent plasma. Hospitalized patients were also more likely to receive additional COVID-19 treatments: 74% of patients received remdesivir, 69% received corticosteroids, and 4% received tocilizumab (Table 1).

### 3.2. Outcomes

The overall mortality rate in our cohort was 11%; of sixty-four patients, seven patients died within 90 days of treatment with passive antibody therapies. The COVID-19 related mortality rate was 4.69%; three deaths were attributed to respiratory failure caused by COVID-19 infection. The majority of patients who died received passive antibody therapies inpatient while hospitalized (*p* = 0.0381). All COVID-19-related deaths occurred in patients who were treated while inpatient (*p* = 0.0178) (Table 1).

In comparison with those who survived, deceased patients were older (70.4 vs. 54.9), and more likely to have additional risk factors for severe COVID-19 disease (*p* = 0.0526). Four of the deceased patients received monoclonal antibodies, while three were treated with COVID-19 convalescent plasma. The deceased patients were more likely to have received additional treatments that represented the standard of care at that time (*p* = 0.0940) (Table 2).

**Figure 2:**
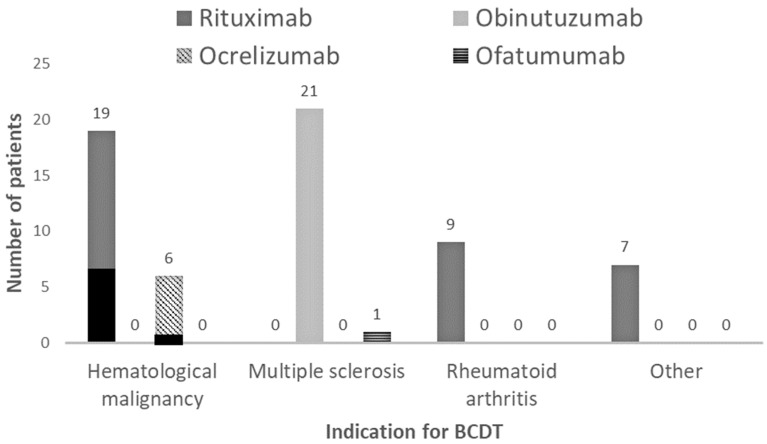
Indications (hematological malignancy, multiple sclerosis, rheumatoid arthritis and others) for B-cell-depleting therapy (BCDT) and type of BCDT (Rituximab, Obinutuzumab, Ocrelizumab, and Ofatumumab) used for each indication. Black areas represent deaths: 6 patients treated with Rituximab and 1 treated with Ocrelizumab died, all with underlying hematological malignancies.

All deceased patients had an underlying hematological malignancy, compared with 33% of survived patients (*p* = 0.0093) (Table 2). Six out of the seven deceased patients were undergoing treatment with rituximab while one was receiving ocrelizumab (Figure 2).

Of the twenty-three patients treated inpatient, five died. Of the five deaths, three were related to complications of COVID-19 infection.

**Figure A1:**
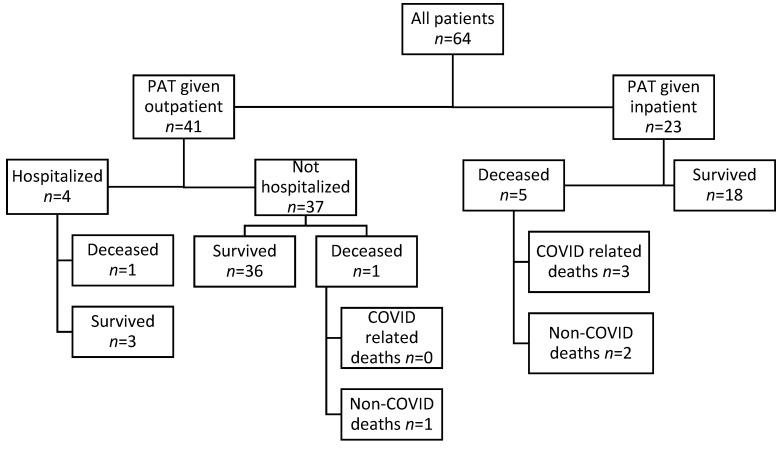
Outcomes (90-day all-cause morbidity and mortality) based on the patient’s location at time of treatment. PAT = passive antibody therapy.

Out of forty-one patients treated in the outpatient setting, two died within 90 days of passive antibody therapy administration from a cause not related to the COVID-19 infection. No COVID-19-related deaths were reported in the patients who received passive antibody therapy in the outpatient setting (Figure A1).

Three of the seven deaths were attributable to respiratory failure caused by COVID-19 infection. All three deceased patients were Caucasian males with multiple comorbidities receiving rituximab for an underlying hematological malignancy. All three of these patients were treated with passive antibody therapy during an inpatient admission for severe COVID-19 disease: two of these patients received COVID-19 convalescent plasma, and one patient received monoclonal antibodies. These patients received additional COVID-19 treatments that represented the standard of care at the time of their hospitalization: all three received a 10-day course of steroids, two received a 5-day course of remdesivir, and two received a single dose of tocilizumab (Table A1).

The other four deaths in our study occurred from causes not related to COVID-19 infection within 90 days of treatment with passive antibody therapy. Two patients who received passive antibody therapy while hospitalized for severe COVID-19 infection recovered and were discharged from the hospital but died at home due to progression of their underlying malignancy at 55 and 65 days after the treatment with passive antibody therapy. Additionally, two patients treated with monoclonal antibodies in the outpatient setting died. One of these patients died at 65 days after the treatment due to progression of their underlying hematologic malignancy. The other patient died at 28 days after passive antibody therapy administration due to an intracranial hemorrhage, after being admitted to the hospital (Table A1).

Of the forty-one patients who were treated in the outpatient setting with passive antibody therapy, four were hospitalized within 90 days. All four of these patients who were later hospitalized were vaccinated at the time of treatment and treated with monoclonal antibodies within seven days of the onset of symptoms. All had an underlying hematological malignancy for which three patients were receiving rituximab and one patient was receiving obinutuzumab. One patient who was admitted to the hospital died from an intracranial hemorrhage 28 days after passive antibody therapies administration. Three patients were admitted to the hospital within 90 days for reasons not related to COVID-19 disease after being fully recovered from COVID-19 infection. They were later discharged in stable condition (Figure A1).

## 4. Discussion

Patients treated with B-cell-depleting therapies have been described to have higher COVID-19-related mortality than matched comparators of patients that have not received B-cell-depleting therapy, with mortality rates ranging between 11% and 32%, and an even higher rate of morbidity as defined through emergency department, hospital, and intensive care unit utilization [21,22,23]. Patients receiving B-cell-depleting agents tend to have longer median hospitalization, intensive care unit stays and days on mechanical ventilation [4]. They also appear to receive more empiric antibiotic therapy and have more complications due to other infections, including bacterial and fungal pathogens [4]. B-cell-depleting agents such as rituximab result in profound B-cell depletion within days of treatment and recovery takes months after discontinuation of B-cell-depleting therapy [4]. This also results in prolonged depletion of memory B-cells, which are an important component of active immunity and long-term protection against antigens. Depletion of B-cells leads to an inability to make effective neutralizing antibodies, which are essential for viral clearance [4,6]. Based on this, theoretically, the use of passive antibody therapies, such as either COVID-19 convalescent plasma or monoclonal antibodies, to treat COVID-19 infection in this patient population would improve disease outcomes and decrease morbidity and mortality rates. However, little is known about the efficacy of passive antibody therapies in immunocompromised patients, much less in those receiving B-cell-depleting therapies, as clinical trials of passive antibody therapies for COVID-19 infection have not generally included the immunocompromised population. In a systematic review and a large meta-analysis that included three randomized controlled trials, the use of COVID-19 convalescent plasma in immunocompromised patients was associated with mortality benefits [12]. Several small cohort studies of patients who received B-cell-depleting therapies and were treated with either COVID-19 convalescent plasma or monoclonal antibodies for COVID-19 infection have suggested more rapid resolution of symptoms and mortality benefit [13,14,15,16]. The mortality rate in our cohort was 11%, with only 4.7% of deaths being attributed to COVID-19, which is much lower than previously reported mortality rates in this patient population. This supports the postulated hypothesis that passive antibody therapies may effectively treat COVID-19 infection in immunosuppressed patients receiving B-cell-depleting therapies.

Clinical trials involving COVID-19 convalescent plasma and monoclonal antibodies indicated that those who benefited most from treatment with passive antibody therapy had mild to moderate illness and received therapy within 5 to 7 days of the onset of symptoms [24,25,26]. Trial results led the Food and Drug Administration to approve these interventions for use in controlled settings under strictly regulated conditions, as defined in the different Emergency Use Authorizations [17,18,19,20]. In our cohort, 85% of those treated as an outpatient received passive antibody therapy within 7 days of symptom onset, compared with only 22% of hospitalized patients (*p* < 0.0001). The fact that there were no deaths attributable to COVID-19 in this group supports the Food and Drug Administration decision to authorize these monoclonal antibodies for treatment of non-hospitalized patients with mild to moderate illness in the early course of the disease.

Studies have shown that vaccination against SARS-CoV-2 in patients with hematologic malignancies was less effective in producing of neutralizing antibodies compared to controls without a hematologic malignancy and active treatment with B-cell-depleting agents further hindered antibody response to vaccination [5]. Some studies suggested additive benefits between COVID-19 vaccination and administration of monoclonal antibodies [27,28]. The fact that 80% of our outpatient cohort was vaccinated may support the presence of such a benefit of COVID-19 monoclonal antibodies to vaccination. However, it is difficult to draw any definitive conclusions about the effect of vaccination in our cohort as we did not have enough non-vaccinated patients treated outpatient to do a comparison with vaccinated patients, nor anti-SARS-CoV-2 antibody status for vaccinated patients to confirm their response to the SARS-CoV-2 vaccine.

Studies of hospitalized COVID-19 patients receiving monoclonal antibody therapy only showed mortality benefit for those who were seronegative at time of hospitalization [29,30,31]. Patients receiving B-cell-depleting therapies with COVID-19 are typically seronegative at time of presentation to hospital; thus, they are a group of patients that may have a mortality benefit from passive COVID-19 antibody therapy. The all-cause mortality rate of patients who received passive antibody therapy while hospitalized for severe COVID-19 disease was 22%, while the COVID-19-related mortality rate was 13%. These findings may suggest a potential benefit from passive antibody therapy in certain hospitalized patients with COVID-19 infection who are receiving B-cell-depleting therapies. It is noticeable that the three patients who were found to be deceased from COVID-19-related respiratory failure were treated within 4, 8 and 11 days of the onset of symptoms, compared with 12 survived patients with severe COVID-19 disease that were treated with passive antibody therapy more than 21 days after symptom onset. We acknowledge that the majority of these patients received other COVID-19 treatments, and we were not able to determine if positive outcomes were directly related to the administration of passive antibody therapy, associated with the use of other therapies or due to the combined effect of all therapies. Further studies are needed to understand which specific subset of hospitalized patients receive maximal benefit from passive antibody therapies.

The disease course in patients receiving B-cell-depleting therapies varies from moderate to severe [32]. The severity of the disease correlates with the B-cell-depleting therapy-induced inability to make effective neutralizing antibodies, which are essential for viral clearance. CD4 and CD8 T-cell responses are unaffected by B-cell-depleting therapies, which may explain why some of these patients have less severe COVID-19 infection, especially if they have been previously vaccinated against COVID-19 and were able to mount some level of active immune response [4,6]. However, strong CD4 and CD8 T-cell responses are not sufficient to control and eradicate infection without the presence of neutralizing antibodies. To assess potential risk factors for severe COVID-19 infection, we evaluated the relationship between the outcome, type of B-cell-depleting therapies, and underlying disease that represented an indication for the use of B-cell-depleting therapies. A study at Cleveland Clinic of over 1600 patients with immune-mediated diseases found that receipt of B-cell-depleting therapy was associated with more severe outcomes [32]. The most extensive retrospective review of the impact of long-term immunosuppressive medication uses on COVID-19 outcomes included over 16,000 immunosuppressed patients and revealed an association between in-hospital death and patients undergoing treatment with rituximab for rheumatological disease and cancer [33]. In our cohort, the majority of deaths occurred in patients treated with rituximab, including all deaths related to COVID-19 and related complications. All deaths occurred in patients with underlying hematological malignancies. All patients who were treated with monoclonal antibodies in the outpatient setting and subsequently hospitalized also had underlying hematological malignancies. Our findings support the postulated relationship between severe COVID-19 infection and rituximab use but also emphasize the central role of underlying comorbidity for which B-cell-depleting therapies is needed in developing severe COVID-19 infection and death. Studies have shown that underlying hematologic malignancy is associated with more severe outcomes in COVID-19 infection compared to the general population [5]. In contrast to the studies that found increased morbidity and mortality rates in patients treated with rituximab for neurological and rheumatological conditions [21,34,35], our findings suggest that patients with non-hematological disorders may have better COVID-19 infection outcomes if treated with passive antibody therapies.

Our study has several limitations. It is a retrospective study containing flaws that cannot be ignored. First, we did not have a control group of patients not receiving B-cell-depleting therapies to compare with our cohort. Furthermore, our sample size of 64 was small and from a single center; thus, it may not be representative of cohorts elsewhere. Another drawback of our study was the relative homogeneity of the study population. Over 90% of our study population was non-Hispanic Caucasian and did not include any persons of Asian descent. Minorities such as African-Americans and Hispanics were underrepresented, and our findings may not be generalizable to these ethnicities. This is especially important, as the COVID-19 pandemic has disproportionately affected Hispanics and African-Americans [36]. Interestingly, all patients treated in the outpatient setting were non-Hispanic Caucasians. This raises concern about health care access for minorities and accentuates the importance of health equity. Due to the retrospective nature of the study, we were not able to evaluate the level of patients’ physical activity that may influence COVID-19 disease outcomes [37]. Data were collected on patients treated over the course of 27 months, during which epidemiological situations and standards of care were in active flux, which would also have had a direct effect on outcomes. We collected data on vaccination status; however, the majority of our patients did not report COVID-19 antibody status; thus, serology was not included in the analysis. Lack of serology data precluded us from making any conclusions about the treatment effect of passive antibody therapy based on serostatus. Studies have demonstrated altered antibody responses to SARS-CoV-2 vaccination in patients receiving B-cell-depleting therapies [4], and data on serology status in vaccinated patients would provide valuable information about the possible additive effect of vaccination and passive antibody therapies. Almost a third of our patients also received treatment with remdesivir and steroids; since we did not do a direct comparison of those who received remdesivir and/or steroids to those who did not, we cannot make any definitive conclusions about the effects that treatment with remdesivir and/or steroids may have had on our cohort.

## 5. Conclusions

Timely administration of passive antibody therapies in the early stages of COVID-19 infection, prior to the development of severe disease, may prove to be beneficial for patients receiving B-cell-depleting therapies. Passive antibody therapies may also be beneficial to certain groups of immunocompromised patients with severe presentation and later in the course of the disease; however, further controlled studies are needed to confirm this and identify patients who would be maximally impacted. Elderly patients with multiple comorbidities, who are actively being treated with B-cell-depleting therapies, particularly rituximab, for an underlying hematological malignancy may be at increased risk for severe COVID-19 disease and poor outcomes from infection, and additional efforts are needed to identify optimal COVID-19 treatment approach for this patient population.

## Figures and Tables

**Table 1:** Patient characteristics and outcomes in the whole cohort and in two groups, inpatient and outpatient, based on the location of passive antibody therapy administration.

	Total *n* = 64 (100%)	Outpatient *n* = 41 (64%*)*	Inpatient *n =* 23 (36%)	*p*-Value
Age, years	56.6	51.4	65.9	
Sex, *n* (%)				0.9738
Male	28 (44)	18 (44)	10 (43)	
Female	36 (56)	23 (56)	13 (56)	
Race, *n* (%)				0.0604
Non-Hispanic Caucasian	61 (95)	41 (100)	20 (87)	
Black or African-American	2 (3)	0(0)	2 (9)	
Hispanic	1 (2)	0(0)	1 (4)	
Vaccination status, *n* (%)				0.0025
Vaccinated	43 (67)	33 (80)	10 (43)	
Unvaccinated	21 (33)	8 (20)	13 (57)	
Indication for BCDT, *n* (%)				0.0002
Hematological malignancy	26 (40)	9 (22)	17 (74)	
Multiple sclerosis	22 (34)	21 (51)	1 (4)	
Rheumatoid arthritis	10 (16)	7 (17)	3 (13)	
Other *	6 (10)	4 (10)	2 (9)	
Type of BCDT, *n* (%)				0.0013
Rituximab	35 (55)	18 (44)	17 (74)	
Obinutuzumab	21 (33)	20 (49)	1 (4)	
Ocrelizumab	7 (11)	2 (5)	5 (22)	
Ofatumumab	1 (1)	1 (2)	0 (0)	
Number of comorbidities, *n* (%)				0.019
Zero	15 (23)	13 (32)	2 (9)	
One	20 (31)	14 (34)	6 (3)	
Two	10 (16)	7 (17)	3 (13)	
Three or more	19 (30)	7 (17)	12 (52)	
Days from the symptom onset till PAT				<0.00001
0–7	40 (63)	35 (85)	5 (22)	
8–14	8 (12)	4 (10)	4 (17)	
14–21	3 (5)	1 (2)	2 (9)	
>21	13 (20)	1 (2)	12 (52)	
Type of PAT, *n* (%)				
mAb	49 (77)	41 (100)	8 (35)	
Casirivimab–imdevimab	31 (48)	25 (4)	8 (35)	
Sotrovimab	13 (20)	13 (32)	0	
Bamlanivimab–etesevimab	4 (6)	2 (5)	0	
Bebtelovimab	1 (2)	1 (2)	0	
CCP	15 (23)	0	15 (65)	
Additional COVID treatments				0.1225
Paxlovid	1 (2)	1 (2)	0	
Evusheld	1 (2)	1 (2)	0	
Remdesivir	21 (33)	4 (1)	17 (74)	
Corticosteroids **	21 (33)	5 (12)	16 (69)	
Tocilizumab	2 (4)	0(0)	2 (8)	
All-cause 90-day mortality	7 (11)	2 (4.88)	5 (21.74)	0.0381
COVID-19-related mortality	3 (4.69)	0(0)	3 (13.04)	0.0178

BCDT = B-cell-depleting therapy. PAT = passive antibody therapy. mAb = monoclonal antibody. CCP = COVID-19 convalescent plasma. * Other indications for BCDT include granulomatosis with polyangiitis (*n* = 3), granulomatous interstitial lung disease (*n* = 1), systemic sclerosis (*n* = 1), systemic lupus (*n* = 1). ** Corticosteroids used for all indications included, not just for COVID-19 treatment.

**Table 2:** Patient characteristics based on the outcomes, 90-day all-cause mortality.

	Total *n* = 64 (100%)	Survived *n* = 57 (89%)	Deceased *n* = 7 (11%)	*p*-Value
Age, years	56.6	54.9	70.4	
Sex, *n*(%)				0.4491
Male	28 (44)	24 (42)	4 (57)	
Female	36 (56)	34 (58)	3 (43)	
Race, *n* (%)				0.5039
Non-Hispanic Caucasian	61 (95)	55 (96)	6 (86)	
Black or African-American	2 (3)	1 (2)	1 (14)	
Hispanic	1 (2)	1 (2)	0 (0)	
Vaccination status, *n* (%)				0.8001
Vaccinated	43 (67)	38 (67)	5 (71)	
Unvaccinated	21 (33)	19 (33)	2 (29)	
Indication for BCDT, *n* (%)				0.0093
Hematological malignancy	26 (40)	19 (33)	7 (100)	
Multiple sclerosis	22 (34)	22 (39)	0 (0)	
Rheumatoid arthritis	10 (16)	10 (17)	0 (0)	
Other	6 (9)	6 (11)	0 (0)	
Type of BCDT, *n* (%)				0.2441
Rituximab	35 (55)	29 (51)	6 (86)	
Obinutuzumab	21 (33)	21 (37)	0 (0)	
Ocrelizumab	7 (11)	6 (11)	1 (14)	
Ofatumumab	1 (1)	1 (1)	0 (0)	
Number of comorbidities, *n* (%)				0.0526
Zero	15 (23)	15 (26)	0 (0)	
One	20 (31)	18 (32)	2 (29)	
Two	10 (16)	10 (17)	0 (0)	
Three or more	19 (30)	14 (25)	5 (71)	
Infusion location, *n* (%)				0.0381
Outpatient	41 (64)	39(68)	2(29)	
Inpatient	23 (36)	18(32)	5(71)	
Type of PAT, *n* (%)				
mAb	49 (77)	45 (79)	4 (57)	0.7708
Casirivimab–imdevimab	31 (48)	28 (49)	3 (43)	
Sotrovimab	13 (20)	12 (21)	1 (4)	
Bamlanivimab–etesevimab	4 (6)	4 (7)	0 (0)	
Bebtelovimab	1 (2)	1 (2)	0 (0)	
CCP	15 (23)	12 (21)	3 (43)	
Additional COVID treatments				0.0940
Paxlovid	1 (2)	1 (2)	0 (0)	
Evusheld	1 (2)	1 (2)	0 (0)	
Remdesivir	21 (33)	17 (30)	4 (57)	
Corticosteroids	21 (33)	17 (30)	4 (57)	
Tocilizumab	2 (4)	0 (0)	2 (28)	

BCDT = B-cell-depleting therapy. PAT = passive antibody therapy. mAb = monoclonal antibody. CCP = COVID-19 convalescent plasma.

## Data Availability

Aggregated data are available upon written request to the corresponding author. Non-aggregated data are not publicly available, as they may contain information that may compromise the privacy of research participants.

## Appendix B

**Table A1:** Characteristics of deceased patients.

Age	Sex	BCDT		PAT		Duration between (days)		Cause of Death
		Indication	Type	Type	Location	Symptom Onset and PAT	PAT and Death	
80	M	CLL	Rituxan	CCP	IP	4	9	Respiratory failure
71	M	DLBCL	Rituxan	CCP	IP	11	13	Respiratory failure
71	M	DLBCL	Rituxan	mAb	IP	8	13	Respiratory failure
71	F	FL	Rituxan	CCP	IP	21	65	Cancer progression
79	F	DLBCL	Rituxan	mAb	OP	1	66	Cancer progression
40	M	CLL	Obinutuzumab	mAb	IP	18	55	Cancer progression
81	F	MCL	Rituxan	mAb	OP	6	28	Intracranial hemorrhage

M = male. F = female. BCDT = B-cell-depleting therapy. PAT = passive antibody therapy. CLL = chronic lymphocytic leukemia. DLBCL = diffuse large B-cell lymphoma. FL = follicular lymphoma. MCL = mantle cell lymphoma. CCP = COVID-19 convalescent plasma. mAb = monoclonal antibodies.
